# Preface to Special Issue: Drug Transporters: Regulation and Roles in Therapeutic Strategies

**DOI:** 10.3390/pharmaceutics16030425

**Published:** 2024-03-20

**Authors:** Guofeng You

**Affiliations:** Department of Pharmaceutics, Rutgers, The State University of New Jersey, Piscataway, NJ 08854, USA; gyou@pharmacy.rutgers.edu

Drug transporters are membrane proteins, mediating, across cell membranes, the absorption, distribution, and excretion of a diverse array of endogenous and exogenous substances such as nutrients, metabolites, toxins, and drugs. Therefore, drug transporters play important roles in human physiology and diseases and in the therapeutic efficacy of many medicines [1,2].

The activity of drug transporters is affected by many factors [3,4]. On the one hand, by directly acting on a transporter molecule, a substance can be an inducer or inhibitor for the activity of the transporter. One the other hand, by acting on the regulatory pathways of the transporter, a substance can modulate gene expression, protein expression, and the cellular localization of the transporter. Key players participating in the regulation of transporters are hormones, protein kinases, nuclear receptors, scaffolding proteins, and disease conditions. This Special Issue presents articles that exemplify these aspects.

In a paper entitled “Molecular Insights to the Structure–Interaction Relationships of Human Proton-Coupled Oligopeptide Transporters (PepTs)”, Luo et al. (contribution 1) described the current understanding of PepTs, focusing on PepT1 and PepT2 which mediate the cellular uptake of dipeptides, tripeptides, and peptide-like drugs. The authors summarized information on the structure–interaction relationships of these transporters and the potential applications of such knowledge in therapeutic optimization and drug development.

Wang et al. (contribution 2) examined the structure–function relationship of liver-specific organic anion transporting polypeptide 1B1 (OATP1B1) in a paper entitled “The Double-Leucine Motifs Affect Internalization, Stability, and Function of Organic Anion Transporting Polypeptide 1B1”. Through site-directed mutagenesis and functional assays, the authors identified three dileucine motifs within the sequence of OATP1B1 to be critical for the trafficking, stability, and substrate recognition of the transporter.

In the paper entitled “Interaction of ALK Inhibitors with Polyspecific Organic Cation Transporters and the Impact of Substrate-Dependent Inhibition on the Prediction of Drug–Drug Interactions”, the authors (contribution 3) dedicated their efforts to investigating the interaction of several tyrosine kinase inhibitors (TKIs) that target aberrant anaplastic lymphoma kinase (ALK), with the polyspecific organic cation transporters (pOCTs) and demonstrated the isoform- and substrate-dependent inhibition potencies of ALK inhibitors on pOCTs. The study highlights the potential drug–drug interactions when using ALK inhibitors in clinic.

The function of transporters not only depends on the “structural fitting” between a transporter and their substrates, but also on how the regulatory machinery/pathways of the transporters are modulated. Liang et al. (contribution 4), in their paper entitled “Chloroquine and Hydroxychloroquine, as Proteasome Inhibitors, Upregulate the Expression and Activity of Organic Anion Transporter 3”, presented the novel finding of chloroquine (CQ) and hydroxychloroquine (HCQ), two well-known anti-malarial drugs, acting as proteasome inhibitors to prevent the degradation of organic anion transporter 3 (OAT3) and therefore as enhancers for OAT3 expression and transport activity.

The paper by Tsang et al. (contribution 5) entitled “Dysregulation of the mRNA Expression of Human Renal Drug Transporters by Proinflammatory Cytokines in Primary Human Proximal Tubular Epithelial Cells” reported the demonstration that proinflammatory cytokines dysregulated (either upregulated or downregulated) the mRNA expression of multiple renal drug transporters, emphasizing the possibility that during inflammation or infections, elevated cytokines can affect alter renal transporter-mediated drug pharmacokinetics. 

Wei et al. (contribution 6) evaluated the effect of exogenous sex and cross-sex hormones on the expression of renal monocarboxylate transporters in a paper entitled “Effect of Sex and Cross-Sex Hormone Treatment on Renal Monocarboxylate-Transporter Expression in Rats”. The authors revealed the altered expression of the transporters in response to these hormones and indicated a future direction in further understanding of the impact of these hormones during puberty on transporter regulation.

Drug transporters play important roles in many pathological conditions. In the paper entitled “Dysregulation of Amino Acid Transporters in a Rat Model of TLR7-Mediated Maternal Immune Activation”, McColl et al. (contribution 7) discovered that the expression of several amino acid transporters was altered in the placenta and fetal brain of the animal model treated with the TLR7 agonist imiquimod and in the human placenta with active infection. This study provided insights into the possible mechanism contributing to neurodevelopmental disorders in humans caused by maternal immune activation during pregnancy.

Verhagen et al. (contribution 8), in a paper entitled “Transporter-Mediated Cellular Distribution of Tyrosine Kinase Inhibitors as a Potential Resistance Mechanism in Chronic Myeloid Leukemia”, summarized current information on the contribution of drug-transporting proteins in the cellular distribution of tyrosine kinase inhibitors used for the treatment of chronic myeloid leukemia. This information can aid in designing more effective and personalized treatment decisions and overcoming drug resistance.

In a paper entitled “Assessing Trans-Inhibition of OATP1B1 and OATP1B3 by Calcineurin and/or PPIase Inhibitors and Global Identification of OATP1B1/3-Associated Proteins”, Powell et al. (contribution 9) reported the trans-inhibitory effects upon OATP1B1 and/or OATP1B3 by the drugs tacrolimus and cyclosporine A (CsA), as well as the CsA analogue SCY-635, in cultured cells. Although the exact mechanism for such inhibition is not known, one possibility is that these drugs interact with OATP1B1/3-associated proteins. Expanding this finding, authors performed proteomics-based screening for protein interactors that may contribute to OATP1B1/3 regulation. Such screening identified various protein kinases, ubiquitin-related enzymes, the tacrolimus (FK506)-binding proteins FKBP5 and FKBP8, and several known regulatory targets of calcineurin, setting the foundation for the further definition of the roles of these proteins in the regulation of OATP1B1/3.

In conclusion, the articles highlighted in this Special Issue, contributed by experts in the drug transport field, offer both broad-range and in-depth insights into the recent advancements in the field of drug transport. We are thankful to all the authors for contributing this cutting-edge information, valuable to researchers in this exciting and rapidly expanding field.
